# AZGP1 Protein Expression in Hormone-Naïve Advanced Prostate Cancer Treated with Primary Androgen Deprivation Therapy

**DOI:** 10.3390/diagnostics10080520

**Published:** 2020-07-27

**Authors:** Mads Dochedahl Winther, Gitte Kristensen, Hein Vincent Stroomberg, Kasper Drimer Berg, Birgitte Grønkær Toft, James D. Brooks, Klaus Brasso, Martin Andreas Røder

**Affiliations:** 1 Copenhagen Prostate Cancer Center, Department of Urology, Rigshospitalet, University of Copenhagen, 2100 Copenhagen, Denmark; madsdochedahlwinther@gmail.com (M.D.W.); hein.vincent.stroomberg@regionh.dk (H.V.S.); kasperdrimerberg@gmail.com (K.D.B.); klaus.brasso@regionh.dk (K.B.); andreasroder@gmail.com (M.A.R.); 2 Department of Pathology, Rigshospitalet, University of Copenhagen, 2100 Copenhagen, Denmark; birgitte.groenkaer.toft@regionh.dk; 3 Department of Urology, Stanford University, Stanford, CA 94305, USA; jbrooks1@stanford.edu

**Keywords:** prostate cancer, AZGP1, biomarker, androgen deprivation therapy

## Abstract

Biomarkers for predicting the risk of castration-resistant prostate cancer (CRPC) in men treated with primary androgen deprivation therapy (ADT) are lacking. We investigated whether Zinc-alpha 2 glycoprotein (AZGP1) expression in the diagnostic biopsies of men with hormone-naïve prostate cancer (PCa) undergoing primary ADT was predictive of the development of CRPC and PCa-specific mortality. The study included 191 patients who commenced ADT from 2000 to 2011. The AZGP1 expression was evaluated using immunohistochemistry and scored as high or low expression. The risks of CRPC and PCa-specific mortality were analyzed using stratified cumulative incidences and a cause-specific COX regression analysis for competing risk assessment. The median follow-up time was 9.8 (IQR: 6.1–12.7) years. In total, 94 and 97 patients presented with low and high AZGP1 expression, respectively. A low AZGP1 expression was found to be associated with a shorter time to CRPC when compared to patients with a high AZGP1 expression (HR: 1.5; 95% CI: 1.0–2.1; *p* = 0.03). However, the multivariable analysis demonstrated no added benefit by adding the AZGP1 expression to prediction models for CRPC. No differences for PCa-specific mortality between the AZGP1 groups were observed. In conclusion, a low AZGP1 expression was associated with a shorter time to CRPC for PCa patients treated with first-line ADT but did not add any predictive information besides well-established clinicopathological variables.

## 1. Introduction

Locally advanced and metastatic prostate cancer (PCa) at the time of diagnosis is associated with a high risk of morbidity and mortality. Recent data suggest that de novo metastatic PCa accounts for more than 60% of men eventually dying from the disease [1]. Androgen deprivation therapy (ADT) remains the primary treatment for men with metastatic PCa. However, recent studies have demonstrated that primary ADT combined with either chemotherapy or newer androgen receptor targeting agents improves the survival in men with metastatic PCa [2,3,4].

Even though PCa initially responds to ADT, the majority of men managed on ADT will eventually progress to castration-resistant prostate cancer (CRPC) [5,6]. For these men, the prognosis is grave, with a median estimated life expectancy of approximately two years [7]. The CRPC disease state is often coupled with debilitating symptoms, such as pain from bone metastases, fatigue, and lower urinary tract symptoms, which decrease the quality of life. Although several of the biological mechanisms of CRPC have been described, there is wide variation in the time from commencing ADT to the development of CRPC. Our lack of knowledge regarding tumor biology beyond the histopathological pattern of growth included in the Gleason score (GS) may explain the current deficiency of the proper prediction of PCa-related outcomes. Immunohistochemistry (IHC), the in situ hybridization of RNA or DNA, and the genomic sequencing of different biomarkers on tissue biopsies are methods routinely used to evaluate tumor biology and the malignant potential of cancers. Increasing our knowledge of primary advanced hormone-naïve PCa by incorporating novel biomarkers could lead to better predictive and prognostic models and thus provide a more personalized approach to treatment.

Zinc-alpha 2 glycoprotein (AZGP1) is an androgen-regulated biomarker in PCa with a potential prognostic impact. A meta-analysis has demonstrated that low AZGP1 expression in tumor tissue is an independent predictor of the time to biochemical failure following radical prostatectomy [8]. Furthermore, it was shown that AZGP1 is an independent predictor of time to CRPC and PCa-specific mortality following radical prostatectomy [8,9,10]. Whether AZGP1 holds prognostic value at other disease stages is, to the best of our knowledge, not known.

In the present study, we investigated the predictive value of AZGP1, which was assessed by applying IHC to tumor tissue samples from men with locally advanced or metastatic PCa commencing ADT as the primary treatment.

## 2. Materials and Methods

### 2.1. Patients and Follow-up Protocol

In the period January 1st 2000 to December 31st 2011, we identified 223 men diagnosed with locally advanced or metastatic PCa, none of whom were considered candidates for curative therapy and all of whom commenced ADT. The patients were followed throughout the period at the out-patient clinic, department of Urology, Rigshospitalet, Denmark. A detailed description of the cohort and follow-up have been presented previously [11]. In brief, the patients were treated with either continuous ADT or the combination of castration with continuous anti-androgen treatment for maximal androgen blockade. Clinical and pathological information was collected retrospectively from medical records and included the age at diagnosis, the tumor, node, and metastasis (TNM) stage, prostate-specific antigen (PSA) at the time of diagnosis, number of biopsies taken, GS on biopsies reevaluated according to the International Society of Urological Pathology (ISUP) 2005 Gleason grading guidelines [12], and primary treatment. Generally, the patients were followed in 3 months intervals with clinical examination and PSA measurements. However, for cases with stable disease, 6 months intervals were allowed for follow-up. The serum testosterone was measured in patients that showed increasing PSA. The follow-up data included the time of progression to CRPC, the time and cause of death if applicable, and any change in the PCa treatment. Prior, 32 patients were excluded due to incomplete follow-up (*n* = 19), missing diagnostic specimens (*n* = 4), or insufficient tissue for further sectioning (*n* = 9).

Our study was conducted adhering to the Reporting recommendations for tumour marker prognostic studies (REMARK) guidelines [13]. The study is approved by the Danish Data Protection Agency (file#2007-58-0015; j.nr. 30-0882) and the Committees on Health Research Ethics in the Capital Region of Denmark (H-2-2012-134).

### 2.2. Endpoints

The endpoints of the study were the risk of CRPC and PCa-specific mortality calculated from the start of ADT. The progression to CRPC was defined in accordance with the European Association of Urology guidelines as the castrate levels of testosterone in combination with biochemical progression or radiological progression [14]. In cases with missing data, CRPC was defined at the time when any treatment approved for CRPC was started.

### 2.3. AZGP1 Immunohistochemistry

**Figure 1:**
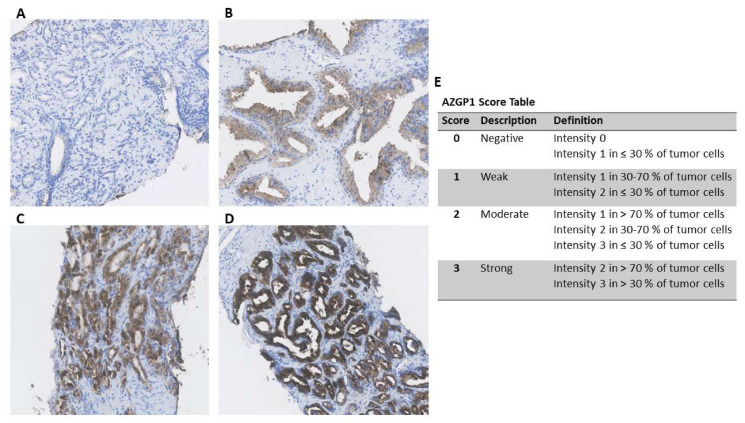
(**A**–**D**) Immunohistochemical Zinc-alpha 2 glycoprotein (AZGP1) staining in representative prostate cancer biopsies displaying negative (**A**), weak (**B**), moderate (**C**), and strong (**D**) staining. (**E**) AZGP1 score table.

Immunohistochemical staining of the diagnostic specimen was performed using a 1:500 dilution of anti-AZGP1 in accordance with a former protocol (HPA012582; Sigma Aldrich, St. Louis, MO, USA) [10]. The stained slides were digitalized using the Hamamatsu Nano ZoomerXR, and evaluation conducted using the Hamamatsu NDP.view V.2.6.12 viewing software. The presence of intracellular AZGP1 immunoreactivity in biopsies was evaluated by one observer (M.D.W.). Each biopsy core was given a score from 0 to 3 based on the fraction of positive tumor cells and the intensity of cytoplasmic staining (Figure 1) [15] using a previously described and standardized approach [8,16]. The immunohistochemical assessment was performed blinded to all clinical endpoints.

As each patient had multiple biopsies, a mean AZGP1 score was calculated to represent the combined immunoreactivity. The AZGP1 score was then dichotomized with the pre-defined cutoff points of ≤ 1.5 (low expression) and > 1.5 (high expression) [15].

### 2.4. Statistics

The baseline clinical characteristics were compared between the AZGP1 groups with the Wilcoxon rank-sum test for continuous variables and ×² test for the categorical variables. The reverse Kaplan–Meier method was used to calculate the median time to follow-up. Cumulative incidences of the study endpoints were analyzed using the Aalen-Johansen method for competing risks, and Gray’s test was used to assess differences between the AZGP1 groups. When analyzing for the risk of CRPC, death before the event was treated as a competing event. Other cause mortality was considered a competing event when analyzing the risk of PCa-specific mortality.

Univariable and multivariable models were performed for the risk of progression to CRPC and PCa-specific mortality and included the AZGP1 expression (high vs low), age at treatment start, log2-transformed PSA, clinical tumor stage, diagnostic GS, and a grouping of metastases. To assess the discriminative ability of the multivariable models with and without the AZGP1 expression, receiver operating characteristic (ROC) curves and area under the curve (AUC) were used for the selected time points. Statistical analyses were performed using R (R Development Core Team, Vienne, Austria). All the tests were two-sided. *p* values < 0.05 were considered statistically significant.

## 3. Results

The final study cohort included 191 patients. The median number of biopsies available for the AZGP1 IHC analysis was 4 (IQR: 3–6) per patient. Ninety-seven (51%) and 94 (49%) patients had high and low AZGP1 expressions, respectively. The baseline characteristics are outlined in Table 1. Patients with a low AZGP1 score had significantly higher clinical stages and worse pathological findings. The median time from diagnosis to the start of ADT was 5 weeks (IQR: 3–12).

**Figure 2:**
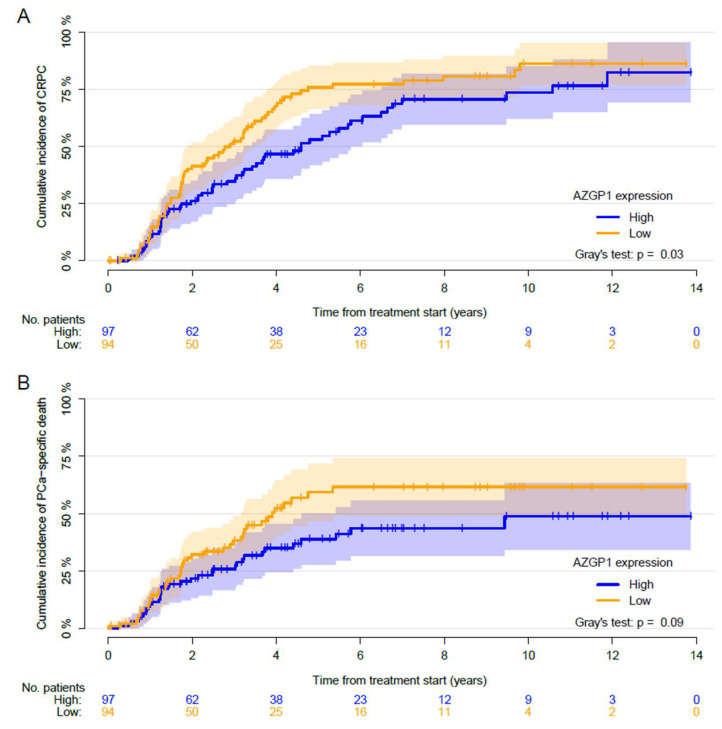
Cumulative incidence of (**A**) progression to castration-resistant prostate cancer (CRPC) and (**B**) prostate cancer (PCa)-specific death after the start of androgen deprivation therapy (ADT) treatment. Competing events are (**A**) death before the progression event and (**B**) death from other causes than PCa. Patients were stratified based on the AZGP1 expression. *p* values for Gray’s test are reported.

With a median follow-up of 9.8 (IQR: 6.1–12.7) years, a total of 125 (65%) patients progressed to CRPC, 41 (21%) patients died of other causes before progressing to CRPC, and 25 (13%) did not progress. The median time to CRPC was 2.9 (95% CI: 1.9–3.5) and 4.6 (95% CI: 3.2–6.1) years in men with low and high AZGP1 expressions, respectively. A significant difference in the cumulative incidence of developing CRPC between the AZGP1 low and high expression group was demonstrated (Gray’s test: *p* = 0.03, Figure 2). A low AZGP1 expression was associated with a higher risk of developing CRPC compared to a high expression in the univariable analysis (HR: 1.5; 95% CI: 1.04–2.1; *p* = 0.03).

**Figure 3:**
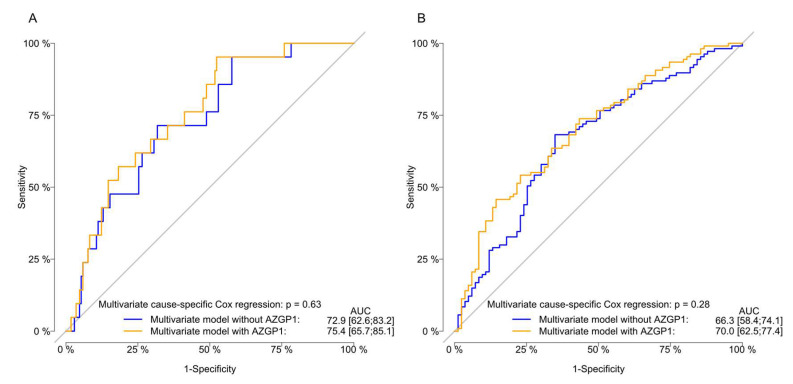
Evaluation of the of the model performance for the multivariate cause-specific Cox regression model for the risk of progression to castration-resistant prostate cancer (CRPC) with receiver-operating characteristic (ROC) curves stratified by AZGP1 expression and omitting the AZGP1 status. Area under the ROC curve (AUC) and *p*-values of the comparative tests are reported one (**A**) and five (**B**) years after the start of ADT.

In a multivariable cause-specific Cox regression analysis (Table 2), only higher PSA was associated with a significantly higher risk of CRPC (HR: 1.2; 95% CI: 1.1–1.4; *p* < 0.0001). The discriminative ability for the prediction of CRPC was not affected by including the AZGP1 expression in the multivariable model. The AUC for including and omitting AZGP1 was 75.4 vs. 72.9 (*p* = 0.6) at 1 year and 70.0 vs. 66.3 after 5 years (*p* = 0.3) (Figure 3).

**Figure 4:**
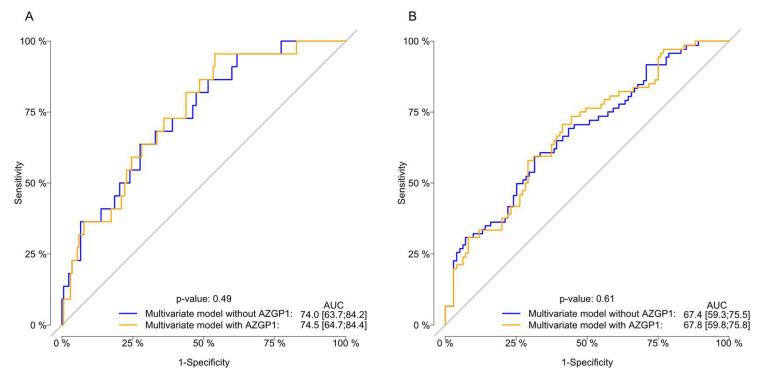
Evaluation of the the model performance for the multivariate cause-specific Cox regression model for the risk of prostate cancer (PCa)-specific death with receiver-operating characteristic (ROC) curves stratified by AZGP1 expression and omitting the AZGP1 status. Area under the ROC curve (AUC) and *p*-values of the comparative tests are reported one (**A**) and five (**B**) years after the start of ADT.

A total of 150 (79%) patients died during the follow-up, 77 (40%) from PCa and 73 (38%) from other causes. The median time from the start of ADT to PCa-specific mortality was 3.9 years (95% CI: 3.2—not reached (NR)) and NR years (95% CI: 4.6-NR) for the AZGP1 low and high expression groups, respectively. The 5-year cumulative incidence of PCa-specific mortality was 48% (95% CI: 40–57), and the cumulative incidence of other cause mortality was 41% (95% CI: 32–50). No differences in the risk of PCa-specific mortality was found when comparing the cumulative incidence for AZGP1 expression, although a trend was found (Gray’s test: *p* = 0.09; Figure 2). After adjustment for other covariates, only PSA remained significantly associated with the risk of PCa-specific mortality (HR: 1.2; 95% CI: 1.1–1.4; *p* < 0.001) (Table 3). Adding the AZGP1 expression to the predictive model did not increase the predictive ability of the model for predicting the PCa-specific mortality at 1 and 5 years (Figure 4). Moreover, AZGP1 loss was not associated with CRPC or PCa-specific mortality when the lowest AZGP1 intensity was used for each patient.

## 4. Discussion

Our hypothesis was that a low AZGP1 expression could predict the early development of resistance to hormone deprivation therapy in hormone-naïve PCa patients. AZGP1 is a secretory protein, and in the prostate AZGP1 secretion is primarily regulated by androgen signaling. The increased expression of AZGP1 mRNA has previously been demonstrated in the LNCaP cell line due to the binding of the androgen receptor to androgen-responsive elements in the promoter region of the AZGP1 gene [17,18,19]. A low AZGP1 expression correlates with a worse pathological tumor stage and higher GS [16,20]. This has led to hypotheses that the loss of or low level of AZGP1 reflects the de-differentiation of the epithelial programs in more aggressive PCa. This de-differentiation could precede the development of disease progression to CRPC by bypassing the androgen receptor pathway [6,18]. Secondly, AZGP1 expression has been found to be associated with poor outcomes in several other cancers, including hepatocellular carcinoma, gastric cancer, and breast cancer [21,22,23].

To the best of our knowledge, the present study is the first to analyze the predictive value of AZGP1 expression in patients with locally advanced or metastatic PCa commencing ADT. We found that patients with a low AZGP1 expression had a significantly higher rate of GS 9–10 and a higher cT-category than patients with a high AZGP1 expression, which is in accordance with previously published studies [8,9,10]. We found that the AZGP1 expression in tumor tissue is predictive of the time to CRPC. However, after adjustment for other clinical and pathological variables, there was no significant discriminatory improvement and no direct association between AZGP1 expression and the development of CRPC. Neither did we find a significant association of AZGP1 expression with the risk of PCa-specific mortality. Thus, the AZGP1 expression did not add any additional predictive information for hormone-naïve PCa patients managed with ADT. There was no predictive value of time to CRPC besides the level of PSA at diagnosis, which may relate to selection bias.

Several studies have demonstrated that a low AZGP1 expression is an independent predictor of a shorter time to biochemical failure and initiating ADT, a shorter time to CRPC, and a greater risk of PCa-specific mortality for men undergoing radical prostatectomy [8,9,10]. A meta-analysis including 11,384 patients from eight different studies demonstrated that a low AZGP1 expression was associated with biochemical failure with a HR of 1.7 following radical prostatectomy [8]. Furthermore, previous research showed that adding AZGP1 expression levels resulted in a significant increase in the AUC from 78 to 83 and c-index from 0.618 to 0.662 in models predicting CRPC following radical prostatectomy [8,10].

One explanation for the apparent lack of predictive information in the later phases of PCa could be biological differences between localized PCa suitable for radical prostatectomy and advanced/metastatic PCa. The recent studies by Kristensen et al. and Zhang et al. consisted of PCa patients undergoing radical prostatectomy predominately for clinically localized disease (98% and 61%, respectively) and with favorable GS (GS ≤ 7 (3 + 4) in 93% and 71%, respectively) [8,9]. In contrast, our population mainly included locally advanced and high-grade GS, likely consistent with more aggressive phenotypes of PCa. Poorly differentiated cancers may have multiple drivers of progression compared to lower-risk tumors, and thus a single biomarker for addressing aggressiveness beyond GS seems overly simplistic. These advanced tumors may have complex and heterogeneous biology and potential mutations in several different pathways driving the disease, which seems too multifaceted for a single IHC marker to be able to evaluate.

A recent study by Burdelski et al. applied certain subdivisions of tumors based on the IHC of several biomarkers, namely AZGP1, ERG, and phosphatase and tensin-homolog (PTEN) [16]. In their radical prostatectomy cohort, they found that a low AZGP1 expression correlated with ERG-positive tumors and tumors with PTEN deletion and that these patients had an increased risk of biochemical failure. The validation of these results in men with advanced PCa is still lacking, but such an approach incorporating multiple biomarkers could provide better insight into the prognostics of AZGP1 in an ADT cohort than looking at AZGP1 by itself as in this study.

Our study has limitations. Since the inclusion of patients into this study, the consensus of primary treatment of metastatic hormone-naïve PCa has changed to include chemotherapy, enzalutamide, or abiraterone acetate in first-line treatment for the selected patients, and thus the primary treatment offered might not reflect the therapeutics used in a more up-to-date population. It is possible that the AZGP1 expression would have performed better in a contemporary cohort. We do not expect that the age of the biopsies could have affected the IHC assessment of AZGP1 expression. Studies have shown a very slow degradation of antigens in paraffin embedding, with slight decay beginning after 15–20 years [24]. Sampling bias due to tumor heterogeneity and/or multiple tumors in the prostate with varying phenotype has been and continues to be a hurdle in PCa research. Significant differences in GS between biopsies and subsequent radical prostatectomy specimens have been established several times [25,26]. With regards to AZGP1, Zhang et al. recently found no correlation between the AZGP1 expression in biopsies and RP specimens, and although the AZGP1 expression level in the RP specimens was predictive for clinical endpoints, the biopsy AZGP1 expression was not [9]. This is somewhat in agreement with our results and, although not possible to confirm, it is likely these technical aspects interfere with the assessment of patients’ AZGP1 expressions. It is possible that the better sampling of the prostate tumor by acquiring more biopsies for IHC analyses than the median of 4 in this study would be necessary to better assess the AZGP1 expression of the hole PCa.

Biomarkers for predicting patients at risk of progression from hormone-naïve to CRPC remain an unmet need in advanced PCa. Currently, androgen receptor variants such as AR-V7 have received most attention, and also as tissue biomarkers. Qu et al. demonstrated that AR-V7 expression in men with newly diagnosed PCa related to the time to CRPC in a multivariate analysis [27]. However, large validation and prospective studies are needed to understand the value of AR variants at diagnosis. A promising study on AR variants and other biomarkers in the treatment of mCRPC and soon de novo metastatic PCa is ongoing [28]. We believe it is unlikely that any single biomarker will predict progression, and we anticipate that panels of biomarkers (such as IHC or transcripts) will improve the predictive performance. Additionally, recent advancements in technologies for analyzing circulating tumor cells (CTC) could have a role by allowing the serial sampling of cancer-derived cells over time. Several groups have reported sequencing analyses of CTCs that demonstrate the complex and heterogeneous tumor biology of metastatic PCa in men treated with ADT and identify gene alterations during the development of CRPC [29,30,31]. Other sequencing approaches, such as the analysis of cell-free DNA for mutations, structural alterations, and methylation, could also be used to understand and predict the transition from the hormone-naïve to the resistant state [32,33]. As sequencing costs continue to drop rapidly, there will be increasing opportunities to transition these technologies from research tools to methods that can be applied in the clinic.

## 5. Conclusions

In this study of AZGP1 expression in PCa specimens from men with locally advanced or metastatic disease managed with first-line ADT, we found that AZGP1 expression was predictive of the development of CRPC. However, the AZGP1 expression did not add further predictive value besides the known predictive clinicopathological features. Furthermore, AZGP1 was not found to be predictive of PCa-specific mortality. Thus, in contrast with early-stage PCa, AZGP1 is unlikely to be a valid biomarker in men commencing ADT.

## Figures and Tables

**Table 1:** Baseline characteristics.

	Study Population (*n* = 191)	High AZGP1 (*n* = 97)	Low AZGP1 (*n* = 94)	*p*-Value
**Age at treatment start, years, median (range)**	70.0 (52.4–89.7)	70.6 (52.4–89.7)	69.5 (52.5–89.1)	0.3
**PSA at treatment start, µg/L, median (IQR)**	62.0 (30.0–202.5)	68.0 (30.0–187.0)	57.5 (30.0–228.0)	0.9
**Clinical tumor category, *n* (%)**				0.05
≤cT2	32 (16.8)	22 (22.7)	10 (10.6)	
cT3a	61 (31.9)	34 (35.1)	27 (28.7)	
cT3b	59 (30.9)	25 (25.8)	34 (36.2)	
cT4	39 (20.4)	16 (16.5)	23 (24.5)	
**Diagnostic Gleason score, *n* (%)**				0.002
≤7	38 (19.9)	28 (28.9)	10 (10.6)	
8	59 (30.9)	31 (32.0)	28 (29.8)	
9–10	94 (49.2)	38 (39.2)	56 (59.6)	
**Lymph node stage, *n* (%)**				0.4
N0	1 (0.5)	0 (0.0)	1 (1.1)	
N1	54 (28.3)	25 (25.8)	29 (30.9)	
Nx	136 (71.2)	72 (74.2)	64 (68.1)	
**Metastasis at diagnosis, *n* (%)**				0.1
M0	70 (36.6)	38 (39.2)	32 (34.0)	
M1	114 (59.7)	53 (54.6)	61 (64.9)	
Mx	7 (3.7)	6 (6.2)	1 (1.1)	
Stage of disease *, *n* (%)				0.3
Locally advanced	40 (20.9)	22 (22.7)	18 (19.1)	
Lymph node metastases only	37 (19.4)	22 (22.7)	15 (16.0)	
Distant metastases	114 (59.7)	53 (54.6)	61 (64.9)	
**Primary ADT treatment, *n* (%)**				0.2
LHRH treatment	176 (92.1)	89 (91.8)	87 (92.6)	
Maximal androgen blockade	8 (4.2)	6 (6.2)	2 (2.1)	
Orchiectomy	7 (3.7)	2 (2.1)	5 (5.3)	

*p*-value: Wilcoxon rank-sum test for continuous variables and ×² test for categorical variables. Abbreviations: IQR: Interquartile range; LHRH: Luteinizing hormone-releasing hormone; TRUS: Transrectal ultrasound; PSA: Prostate-specific antigen. * Locally advanced: N0/Nx and M0/Mx; Lymph node metastases only: N1 and M0/Mx; Distant metastases: M1 and N0/Nx/N1.

**Table 2:** Uni- and multivariable cause-specific Cox proportional hazards of the risk of CRPC.

	Univariable Analysis		Multivariable Analysis	
	HR (95% CI)	*p* Value	HR (95% CI)	*p* Value
**AZGP1 expression**				
High	REF		REF	
Low	1.5 (1.0–2.1)	0.03	1.3 (0.9–1.9)	0.2
**Age at treatment start**	1.0 (1.0–1.02)	0.5	1.0 (1.0–1.02)	0.6
**PSA for 2-fold diff.**	1.2 (1.1–1.4)	<0.0001	1.2 (1.1–1.4)	<0.0001
**Clinical tumor category**				
≤cT2	REF		REF	
cT3a	1.2 (1.0–8.4)	0.5	0.9 (0.5–1.6)	0.7
cT3b	2.0 (0.6–6.4)	0.2	1.0 (0.5–1.7)	0.9
cT4	2.9 (0.9–9.6)	0.07	1.1 (0.5–2.0)	0.9
**Diagnostic Gleason score**				
≤7	REF		REF	
8	1.4 (0.8–2.4)	0.2	1.1 (0.6–1.8)	0.9
9–10	1.8 (1.1–2.9)	0.02	1.5 (0.9–2.5)	0.1
Stage of disease *				
Locally advanced	REF		REF	
Lymph node metastases only	0.8 (0.7–1.8)	0.4	1.1 (0.6–2.0)	0.8
Distant metastases	1.2 (0.5–1.4)	0.5	1.0 (0.6–1.6)	1.0

Abbreviations: CI: Confidence interval; CRPC: Castration-resistant prostate cancer; HR: Hazard ratio; PSA: Prostate-specific antigen. * Locally advanced: N0/Nx and M0/Mx; Lymph node metastases only: N1 and M0/Mx; Distant metastases: M1 and N0/Nx/N1.

**Table 3:** Uni- and multivariable cause-specific Cox proportional hazards of the risk of PCa-specific mortality.

	Univariable Analysis		Multivariable Analysis	
	HR (95% CI)	*p* Value	HR (95% CI)	*p* Value
**AZGP1 expression**				
High	REF		REF	
Low	1.5 (1.0–2.4)	0.06	1.2 (0.7–1.9)	0.6
**Age at treatment start**	1.0 (1.0–1.0)	0.6	1.0 (1.0–1.0)	0.5
**PSA for 2-fold diff.**	1.2 (1.1–1.4)	<0.0001	1.2 (1.1–1.4)	0.001
**Clinical tumor category**				
≤cT2	REF		REF	
cT3a	1.6 (0.7–3.4)	0.3	1.1 (0.5–2.6)	0.8
cT3b	2.3 (1.1–5.0)	0.03	1.6 (0.7–3.5)	0.3
cT4	2.5 (1.1–5.6)	0.02	1.2 (0.5–3.0)	0.7
**Diagnostic Gleason score**				
≤7	REF		REF	
8	1.5 (0.7–2.9)	0.3	0.9 (0.4–1.9)	0.8
9–10	2.0 (1.1–3.8)	0.0	1.5 (0.7–2.8)	0.3
Stage of disease *				
Locally advanced	REF		REF	
Lymph node metastases only	0.7 (0.3–1.6)	0.4	0.9 (0.4–2.1)	0.8
Distant metastases	1.5 (0.9–2.8)	0.2	1.3 (0.7–2.4)	0.5

Abbreviations: CI: Confidence interval; HR: Hazard ratio; PCa: Prostate cancer; PSA: Prostate-specific antigen. * Locally advanced: N0/Nx and M0/Mx; Lymph node metastases only: N1 and M0/Mx; Distant metastases: M1 and N0/Nx/N1.
